# Postnatal care for newborns in Bangladesh: The importance of health–related factors and location

**DOI:** 10.7189/jogh.07.020507

**Published:** 2017-12

**Authors:** Kavita Singh, Paul Brodish, Mahbub Elahi Chowdhury, Taposh Kumar Biswas, Eunsoo Timothy Kim, Christine Godwin, Allisyn Moran

**Affiliations:** 1 MEASURE Evaluation/Carolina Population Center, University of North Carolina at Chapel Hill, Chapel Hill, North Carolina, USA; 2 Department of Maternal and Child Health, Gillings School of Global Public Health, University of North Carolina at Chapel Hill, Chapel Hill, North Carolina, USA; 3 International Centre for Diarrhoeal Disease Research, Bangladesh (icddr,b), Dhaka, Bangladesh; 4 Global Health Fellows Program II, United States Agency for International Development (USAID), Washington, D.C., USA, and Abuja, Nigeria

## Abstract

**Background:**

Bangladesh achieved Millennium Development Goal 4, a two thirds reduction in under–five mortality from 1990 to 2015. However neonatal mortality remains high, and neonatal deaths now account for 62% of under–five deaths in Bangladesh. The objective of this paper is to understand which newborns in Bangladesh are receiving postnatal care (PNC), a set of interventions with the potential to reduce neonatal mortality.

**Methods:**

Using data from the Bangladesh Maternal Mortality Survey (BMMS) 2010 we conducted logistic regression analysis to understand what socio–economic and health–related factors were associated with early postnatal care (PNC) by day 2 and PNC by day 7. Key variables studied were maternal complications (during pregnancy, delivery or after delivery) and contact with the health care system (receipt of any antenatal care, place of delivery and type of delivery attendant). Using data from the BMMS 2010 and an Emergency Obstetric and Neonatal Care (EmONC) 2012 needs assessment, we also presented descriptive maps of PNC coverage overlaid with neonatal mortality rates.

**Results:**

There were several significant findings from the regression analysis. Newborns of mothers having a skilled delivery were significantly more likely to receive PNC (Day 7: OR = 2.16, 95% confidence interval (CI) 1.81, 2.58; Day 2: OR = 2.11, 95% 95% CI 1.76). Newborns of mothers who reported a complication were also significantly more likely to receive PNC with odds ratios varying between 1.3 and 1.6 for complications at the different points along the continuum of care. Urban residence and greater wealth were also significantly associated with PNC. The maps provided visual images of wide variation in PNC coverage and indicated that districts with the highest PNC coverage, did not necessarily have the lowest neonatal mortality rates.

**Conclusion:**

Newborns of mothers who had a skilled delivery or who experienced a complication were more likely to receive PNC than newborns of mothers with a home delivery or who did not report a complication. Given that the majority of women in Bangladesh have a home delivery, strategies are needed to reach their newborns with PNC. Greater focus is also needed to reach poor women in rural areas. Engaging community health workers to conduct home PNC visits may be an interim strategy as Bangladesh strives to increase skilled delivery coverage.

The prevention of neonatal mortality has become a global priority because of the high mortality experienced by newborns and the difficulty in achieving improvements in their survival. Neonatal mortality now accounts for 45% of under–five mortality which translates to 2.8 million deaths within the first 28 days of life. On a global level neonatal mortality rates have declined from 33 to 19 deaths per 1000 live births (from 1990 to 2015), but this 47% reduction is much less than the 58% reduction seen in deaths among post–neonatal children under–five [1]. In addition, an estimated 2.6 million stillbirths occur each year, though only a fraction of these deaths are recorded in vital registration systems [2–4]. An estimated 46% of stillbirths are intrapartum or “fresh” indicating that the fetus died after the onset of labor and perhaps could have been saved with appropriate interventions at delivery [3].

*Every Newborn* is an action plan focused on enabling countries to prevent neonatal deaths and stillbirths [4]. The plan emphasizes the critical periods of labor, birth and the first week of life as time points when interventions can achieve maximum impact on saving newborn lives. The plan is an extension of the United Nation’s *Every Woman Every Child* movement and includes a vision, goals, strategies, and priorities for reducing newborn and stillbirth deaths. To achieve these goals, *Every Newborn* lays out key strategic objectives: 1. Improving care at birth, 2. Improving the quality and equity of maternal and newborn care, 3. Reaching every woman and newborn and achieving impact at scale, 4. Harnessing the power of parents, families and communities, and 5. Counting every newborn through measurement, program–tracking and accountability. Referral and follow–up care for low birth weight and sick newborns is also crucial given that prematurity and low birth weight are major predicators of neonatal mortality.

A continuum of services is needed to enhance newborn survival. Essential newborn care (ENC) involves care soon after birth and includes hygienic care, thermal control, support for breastfeeding and resuscitation with bag and mask, if needed [4]. Such interventions can address the main causes of neonatal mortality such as intrapartum related birth asphyxia and complications due to prematurity and low birth weight, which account for more than half of neonatal deaths [5]. Complementing essential newborn care is postnatal care (PNC) for the newborn, a package of interventions delivered after birth that includes the promotion of immediate and exclusive breastfeeding (for children less than 6 months of age), hand–washing, examination of mother and child for danger signs and appropriate referral for medical care [6]. Interventions provided as part of PNC can prevent some newborn complications such as sepsis, meningitis, pneumonia and diarrhea. PNC could be a means of providing follow–up care to newborns who were born premature and/or of low birth weight and provides an opportunity to check all newborns for illnesses that may have arisen since delivery [4].

Under–five mortality in Bangladesh has been steadily declining from 144 deaths per 1000 live births to 38 deaths per 1000 live births in the period between 1990 and 2015 [1]. Though Bangladesh laudably achieved the Millennium Development Goal (MDG) 4 target of a two–thirds reduction in under–five mortality, the burden of neonatal mortality continues to remain a concern. Neonatal mortality also declined from 63/1000 to 23/1000 during the same time period, but the magnitude of the decline was not as great as for under–five mortality. The proportion of neonatal deaths out of all under–five deaths actually increased from 44% to 62% from 1990 to 2015 [1].

Promotion of PNC has been emphasized in the National Neonatal Health Strategy and Guidelines (NNHS) of Bangladesh, and PNC is provided free of charge at government health facilities. PNC is provided both at health facilities and during home visits by community health workers in efforts to make the service accessible from the community to tertiary level. During home visits community health workers focus on the i) promotion of newborn care (early/exclusive breastfeeding, warmth, hygiene); ii) promotion of nutrition & family planning counseling to mothers; iii) providing information about danger signs of both mother and newborn; iv) Identification of danger signs in newborn and referral; v) support for breastfeeding; and vi) care of low birth weight infant (feeding, skin–to–skin contact) [7,8].

Studies in Bangladesh have found that socioeconomic factors such as education [9,10] and wealth have an influence on PNC coverage for newborns [11–16]. A study by Anwar et al. 2008 found that having at least one ANC visit [14] was associated with higher utilization of PNC, suggesting that prior contact with the health care system may be important. A qualitative study by Syed et al. 2008 found that mothers did not perceive PNC for themselves or their babies to be of much value unless they had a complication or their newborn was sick. The same study found that knowledge of maternal and newborn complications was often limited and initial care–seeking was often with a non–formal provider [17]. No quantitative studies in Bangladesh have looked at the role of complications on receipt of PNC for newborns.

The main aim of this study is to delve deeper into the question of which newborns in Bangladesh are receiving PNC by exploring not just socio–economic factors but also health–related factors, including maternal complications and contact with the health system. Our aim is to understand whether these factors, which have not been extensively studied in the literature, are associated with PNC. A secondary aim is to use maps to descriptively present geographic variability in PNC coverage and neonatal mortality. Maps can be a useful means to pinpoint geographic areas which need more programmatic focus.

## METHODS

### Data and sample

Data came from the 2010 Bangladesh Maternal Mortality Survey (BMMS), a large–scale survey of 175 000 households [18]. The BMMS employed a multi–stage selection procedure designed to provide representative samples for maternal mortality at the national level and representative estimates at the national, urban/rural, divisional, and district levels for most other indicators. The BMMS Women’s Long Questionnaire, which collected socio–economic and health–related information from approximately 62 000 ever–married women aged 13–49, was used in this study. We also obtained information on household wealth from the BMMS Household Questionnaire. Because the primary outcome of interest was PNC for the most recent birth, we restricted the sample to those women who had a live birth in the past six years for a total sample size of 25 014 mothers.

Data for the maps came from the BMMS 2010 as well as from a 2012 Needs Assessment of Emergency Obstetric and Newborn Care (EmONC) [19]. The BMMS 2010 provided the PNC data and population–level neonatal mortality estimates for all districts while the EmONC assessment provided data on facility–level neonatal mortality for 24 of the 64 districts of Bangladesh.

This study was reviewed by the Institutional Review Board (IRB) at the University of North Carolina at Chapel Hill and was exempted from needing ethics review approval because of the secondary nature of the analysis.

### Descriptive and regression analyses

#### Outcomes

We calculated simple weighted descriptive statistics and chi–square analyses on all predictor and demographic variables, comparing women who reported receiving PNC on or before day 7 and on or before day 2 (early PNC) to those who did not. The sample for early PNC is a subset of the larger PNC on or before day 7 sample. Early PNC was defined as within a day for facility births and within two days for non–facility births. The WHO indicates that PNC should be given to newborns within 24 hours for both facility births and as soon as possible for non–facility births [6,20]. We therefore included day 2 as relevant for early PNC for non–facility births.

#### Key independent variables

Our key predictor variables were focused on access to maternal health services and the presence of complications. In terms of maternal health services we included receipt of ANC, type of delivery attendant (Skilled Birth Attendant (SBA) or non–SBA), place of delivery (facility vs non–facility). A SBA was defined according to the World Health Organization’s definition as “an accredited health professional – such as a midwife, doctor or nurse – who has been educated and trained to proficiency in the skills needed to manage normal (uncomplicated) pregnancies, childbirth and the immediate postnatal period, and in the identification, management and referral of complications in women and newborns” [21]. We, thus, defined a SBA as an accredited doctor, nurse or midwife. All others including traditional birth attendants (TBAs) were defined as non–SBAs. We looked at both the type of delivery attendant and place of delivery because Bangladesh promotes a strategy of home deliveries by SBAs when facility delivery is not feasible [22,23]. Complications reported by the mother at labor, delivery and after delivery were also key measures in our analysis. Though the BMMS included questions on timing of complication, questions on specific types of complications were not included. We were not able to include four or more ANC visits and low birth weight in our analysis because of a large amount of missing data, and data on neonatal complications were not available.

#### Regression analysis

We performed weighted logistic regression models predicting receipt of the PNC outcomes, controlling for maternal age, parity, highest level of education, urban residence, marital status, and wealth quintile. The wealth index was constructed from data on ownership of household items including bathroom facilities, roofing, and flooring. Each asset was assigned a weight (factor score) generated through principle components analysis. Each household’s scores were then summed; individuals were ranked according to the total score of the household in which they resided [18]). All analyses were performed using Stata v. 14.

#### Maps and chi–square comparisons

Descriptive maps of PNC by day 7 and early PNC were interposed with data on population–level neonatal mortality and facility–level neonatal mortality to present subnational level variation in PNC coverage and neonatal mortality. We also performed χ^2^ analyses of the key independent variables and PNC with neonatal mortality. Studying associations between PNC and neonatal mortality, however, has its limitations. The data do not allow for a determination of whether or not deaths on the first day of life occurred to newborns before they were even eligible for PNC [9]. For example, there could be some left censoring in that some newborns might have died within minutes of birth before they could have received a PNC check, but the data does not disaggregate deaths on the first day into hours or minutes.

## RESULTS

**Table 1** reports sample characteristics for PNC by day 7, while **Table 2** does the same for early PNC. Given that early PNC is a subset of the PNC by day 7 outcome, the number of women who received early PNC is smaller than PNC by day 7 (7461 vs 8258). Thirty–three percent of respondents reported receipt of PNC by day 7, while 30% reported early PNC. For every characteristic except marital status, there were differences between women receiving PNC by day 7 and not receiving PNC by day 7. For example, women who reported contact with the health system in terms of ANC, facility delivery or delivery with a SBA were more likely to report PNC by day 7. Women who reported a complication during pregnancy, delivery or after delivery as well as urban, wealthier and more educated were also more likely to indicate their newborns received PNC by day 7. Results were similar when comparing women receiving early PNC to those who did not (**Table 2**).

**Table 1:** Sample characteristics by postnatal care on or before day 7 (n = 25 014)

	No PNC by day 7		PNC by day 7		
**Characteristic**	**n**	**(%)**	**n**	**(%)**	***P***
Total	16 756	67.0	8258	33.0	
**Predictor variables**					
Delivery:					
- Unskilled	15 307	93.3	3489	46.0	
- Skilled	1449	6.7	4769	54.0	<0.001
Home delivery:					
- No	1078	5.2	4622	52.7	
- Yes	15 678	94.8	3636	47.3	<0.001
Any antenatal care (ANC):					
- No	6615	40.5	1135	14.8	
- Yes	10 141	59.5	7123	85.2	<0.001
Reported complications during pregnancy:					
- No	11 190	66.5	4158	49.7	
- Yes	5566	33.5	4100	50.3	<0.001
Reported complications during delivery:					
- No	13 099	78.0	5209	62.5	
- Yes	3657	22.0	3049	37.5	<0.001
Reported complications after delivery:					
- No	13 818	82.8	6270	75.9	
- Yes	2938	17.2	1988	24.1	<0.001
**Demographic characteristics**					
Maternal age (years):					
- 13 to 19	1950	11.7	914	11.5	
- 20 to 24	5363	32.6	2938	36.2	
- 25 to 29	4782	28.3	2329	27.9	
- 30 to 34	2677	15.9	1268	14.8	
- 35 to 39	1324	7.6	574	6.7	
- 40 to 44	485	2.9	179	2.1	
- 45 to 49	175	1.0	56	0.7	<0.001
Highest level of education (class):					
- None	4964	30.2	1269	16.0	
- 1 to 5	5658	33.3	2031	25.5	
- 6 to 8	3402	20.7	1789	22.6	
- 9+	2732	15.8	3169	35.9	<0.001
Urban residence:					
- No	10 773	81.9	3955	64.4	
- Yes	5983	18.1	4303	35.6	<0.001
Religion:					
- Islam	15 349	92.4	7334	89.8	
- Hindu/other	1407	7.6	924	10.2	<0.001
Marital status:					
- Currently not married	324	1.8	127	1.5	
- Currently married	16 432	98.2	8131	98.5	0.199
Wealth index quintile:					
- Poorest	4234	26.4	790	10.8	
- Poorer	3702	22.8	964	12.5	
- Middle	3522	21.5	1384	18.0	
- Richer	3047	17.7	1878	22.7	
- Richest	2251	11.6	3242	35.9	<0.001

**Table 2:** Sample characteristics by postnatal care on or before day 2 (early PNC, n = 25 014)

	No PNC by day 2		PNC by day 2		
**Characteristic**	**n**	**%**	**n**	**%**	***P***
Total	17 553	70.2	7461	29.8	
**Predictor variables**					
Delivery:					
- Unskilled	15 898	92.6	2898	42.6	
- Skilled	1655	7.4	4563	57.4	<0.001
Home delivery:					
- No	1247	5.7	4453	56.4	
- Yes	16 306	94.3	3008	43.6	<0.001
Any antenatal care (ANC):					
- No	6786	39.7	964	14.0	
- Yes	10 767	60.3	6497	86.0	
Reported complications during pregnancy:					
- No	11 584	65.7	3764	49.8	
- Yes	5969	34.3	3697	50.2	<0.001
Reported complications during delivery:					
- No	13 637	77.5	4671	61.9	
- Yes	3916	22.5	2790	38.1	<0.001
Reported complications after delivery:					
- No	14 402	82.4	5686	76.2	
- Yes	3151	17.6	1775	23.8	<0.001
**Demographic characteristics**					
Maternal age (years):					
- 13 to 19	2036	11.7	828	11.5	
- 20 to 24	5646	32.7	2655	36.4	
- 25 to 29	4999	28.3	2112	28.0	
- 30 to 34	2816	16.0	1129	14.5	
- 35 to 39	1371	7.5	527	6.9	
- 40 to 44	502	2.8	162	2.1	
- 45 to 49	183	1.0	48	0.7	<0.001
Highest level of education (class):					
- None	5122	29.8	1111	15.5	
- 1 to 5	5924	33.3	1765	24.7	
- 6 to 8	3567	20.8	1624	22.6	
- 9+	2940	16.1	2961	37.2	<0.001
Urban residence:					
- No	11 226	81.6	3502	63.3	
- Yes	6327	18.4	3959	36.7	<0.001
Religion:					
- Islam	16 072	92.3	6611	89.6	
- Hindu/other	1481	7.7	850	10.4	<0.001
Marital status:					
- Currently not married	340	1.8	111	1.5	
- Currently married	17 213	98.2	7350	98.5	0.2028
Wealth index quintile:					
- Poorest	4357	26.0	667	10.1	
- Poorer	3835	22.6	831	11.9	
- Middle	3697	21.6	1209	17.5	
- Richer	3213	17.9	1712	22.9	
- Richest	2451	12.0	3042	37.5	<0.001

**Table 3** shows results from the logistic regressions of PNC by day 7 and by day 2. Confirming some of the findings shown in **Tables 1 **and** 2**, controlling for maternal demographic characteristics, having had a skilled delivery significantly increased the odds of reporting PNC by day 7 and by day 2 by over 2.1 times (odds ratio (OR) = 2.16, 95% confidence interval (CI) 1.81, 2.58; OR = 2.11, 95% CI 1.76, 2.54, respectively). Having had a home delivery significantly decreased the likelihood of PNC by about 85% (OR = 0.15, 95% CI 0.12, 0.18; OR = 0.14, 95% CI 0.12, 0.17, respectively). Reporting complications during pregnancy, delivery and after delivery significantly increased the odds of reporting PNC by between 1.3 and 1.6 times. Urban residence significantly increased the odds of reporting PNC by day 7 or by day 2 by about 1.4 times (OR = 1.38, 95% CI 1.22, 1.55; OR = 1.40, 95% CI 1.23, 1.59, respectively). Being in the top three wealth quintiles also increased the odds of reporting PNC by day 7 and by day 2 by between 1.5 and 2.3 times.

**Table 3:** Logistic regressions of postnatal care by day 7 and by day 2 on predictor variables (n = 25 014)

	PNC by day 7			PNC by day 2		
**Characteristic**	**Odds ratio**	**95% CI**	***P***	**Odds ratio**	**95% CI**	***P***
**Predictor variables**						
Skilled delivery	2.16	1.81–2.58	<0.001	2.11	1.76–2.54	<0.001
Home delivery	0.15	0.12–0.18	<0.001	0.14	0.12–0.17	<0.001
Any antenatal care (ANC)	1.74	1.58–1.92	<0.001	1.71	1.54–1.89	<0.001
Complications during pregnancy	1.60	1.45–1.73	<0.001	1.49	1.36–1.64	<0.001
Complications at delivery	1.48	1.34–1.63	<0.001	1.53	1.38–1.69	<0.001
Complications after delivery	1.36	1.22–1.51	<0.001	1.32	1.18–1.48	<0.001
**Demographic characteristics**						
Maternal age (years):						
- 13 to 19	1.00					
- 20 to 24	1.12	0.98–1.27	0.110	1.10	0.96–1.27	0.172
- 25 to 29	1.07	0.91–1.25	0.431	1.06	0.90–1.26	0.478
- 30 to 34	1.02	0.84–1.23	0.856	0.98	0.81–1.19	0.841
- to 39	1.11	0.89–1.39	0.362	1.18	0.93–1.50	0.184
- 40 to 44	1.13	0.84–1.52	0.403	1.19	0.87–1.61	0.272
- to 49	1.52	1.01–2.29	0.047	1.52	0.98–2.37	0.060
Parity:						
- 1	1.00					
- 2–3	0.91	0.82–1.01	0.069	0.92	0.82–1.02	0.111
- 4+	0.94	0.80–1.11	0.450	0.91	0.76–1.08	0.278
Highest level of education (class):						
- None	1.00					
- 1 to 5	1.10	0.98–1.23	0.091	1.06	0.94–1.20	0.372
- 6 to 8	1.08	0.94–1.23	0.277	1.05	0.91–1.22	0.492
- 9+	1.14	0.99–1.31	0.073	1.11	0.95–1.29	0.174
Urban residence:						
- No	1.00					
- Yes	1.38	1.22–1.55	<0.001	1.40	1.23–1.59	<0.001
Religion:						
- Islam	1.00					
- Hindu/other	1.09	0.93–1.28	0.284	1.07	0.91–1.27	0.412
Marital status:						
- Currently not married	1.00					
- Currently married	1.04	0.77–1.41	0.791	1.04	0.76–1.44	0.793
Wealth index quintile:						
- Poorest	1.00					
- Poorer	1.15	1.00–1.32	0.058	1.14	0.98–1.32	0.090
- Middle	1.47	1.29–1.68	<0.001	1.46	1.27–1.69	<0.001
- Richer	1.65	1.42–1.91	<0.001	1.68	1.43–1.97	<0.001
- Richest	2.32	1.97–2.74	<0.001	2.35	1.97–2.80	<0.001
Constant	0.45	0.31–0.66	<0.001	0.40	0.27–0.60	<0.001

**Figure 1:**
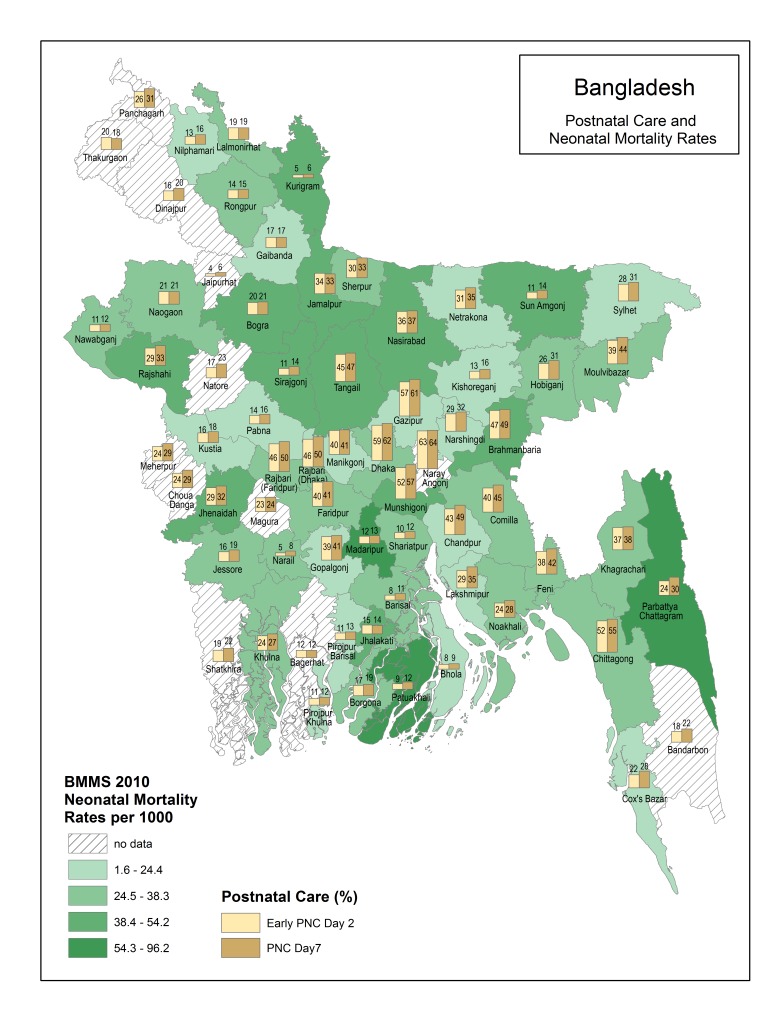
Postnatal care (PNC) and population–level neonatal mortality by district.

**Figure 2:**
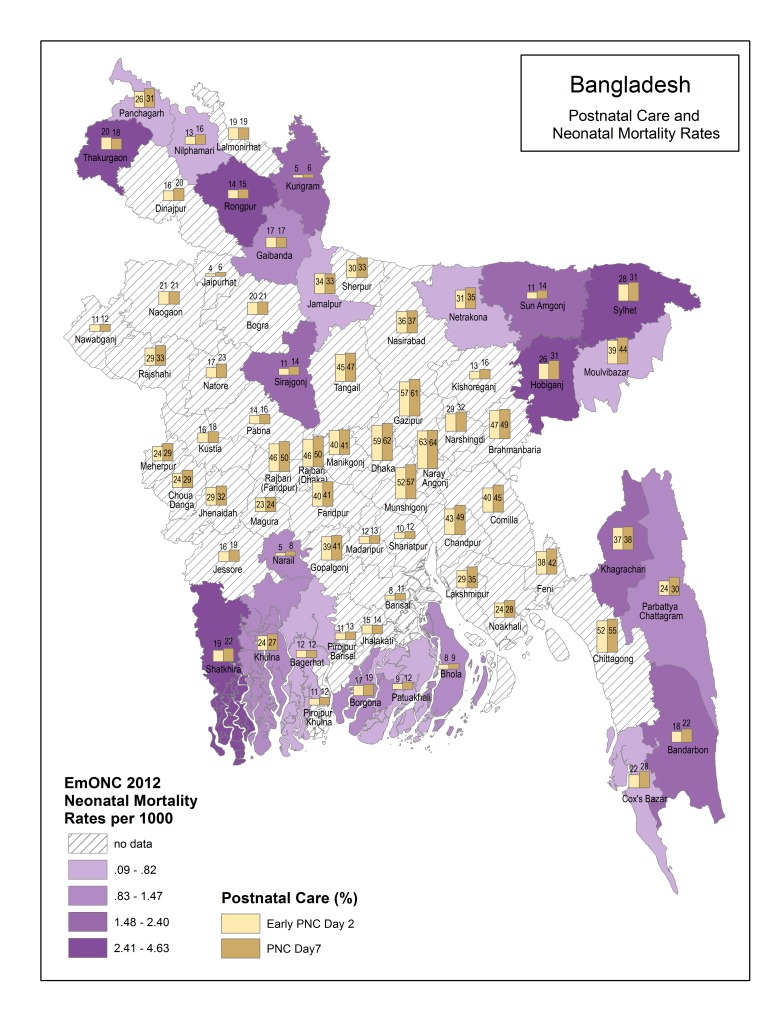
Postnatal care (PNC) and facility–level neonatal mortality by district.

**Figure 3:**
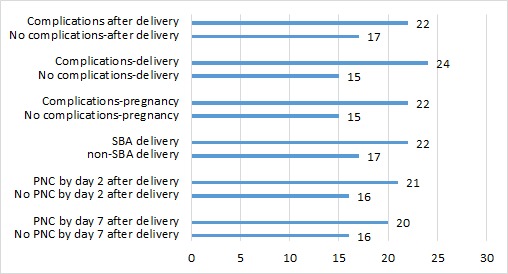
Bivariate associations between selection variables and neonatal mortality (per 1000). Note: All comparisons are significant at *P* < 0.05 or *P* < 0.0001 except for PNC by Day 7 (*P* = 0.0635). PNC – postnatal care, SBA – skilled birth attendant.

**Figures 1 **and** 2** present descriptive maps of PNC coverage interposed with population–level neonatal mortality (**Figure 1**) and facility–level neonatal mortality (**Figure 2**). Overall, there is wide variation in the PNC (from 4% to 64%) and population–level neonatal mortality rates (from 1.6 per 1000 to 96.2 per 1000) within Bangladesh. Districts with the lowest neonatal mortality do not always have the highest PNC coverage and vice versa. Chi–square statistics for key independent variables and PNC with neonatal mortality indicate significantly higher mortality for newborns receiving PNC by day 2 and for newborns of mothers having a complication and a skilled delivery. These results are presented in **Figure 3**.

## DISCUSSION

PNC is a package of interventions intended for all newborns and has both a preventative focus and curative focus. In our study of PNC in Bangladesh we find that newborns whose mothers had a facility delivery or who had a complication were likely to have a PNC check than newborns of mothers who delivered at home or did not have a complication. Given that 79% of women in our sample had a home birth, efforts are needed to ensure all newborns are reached.

PNC for newborns may not always be perceived to be necessary by mothers and their families in Bangladesh [17]), unless there is a complication or the newborn appears sick. Education of families on the importance of PNC as a preventative service can lead to improvements in newborn health. Early treatment for illnesses such as pneumonia is crucial, and PNC offers an opportunity for health workers to look for signs of illnesses which may be missed by family members.

Traditional practices may prevent many mothers from leaving their homes for up to 40 days after delivery [24,25]. During this period of isolation, mothers are considered to be in a state of impurity and vulnerability to evil spirits [25]. Mothers often sleep on thin mats on the floor with their newborns to minimize the spread of pollution to others and protect themselves from spiritual attacks [16,25]. Husbands and mothers–in–law may also serve as gatekeepers to ensure minimal contact with outsiders during this period [25–28]. In light of these cultural issues, training community health workers to provide PNC for mothers and newborns at home, has surfaced as an interim solution to increasing the coverage of PNC [24,29,30]. Community health workers have the ability to gain the trust and support of mothers, fathers and mothers–in–law. They can engage these individuals on discussions concerning newborn care and any harmful traditional practices. For example, if newborns are sleeping on thin mats, community health workers can educate families on the risks of hypothermia. Studies conducted in rural Bangladesh have documented that community health workers are capable of correctly identifying sick newborns with a 6–sign or 7–sign clinical algorithm during their routine surveillance of newborns at home [28,31]. Home visits from community health workers by day 2 have been shown to reduce neonatal mortality [32]. Home visits have also been shown to be an effective means to assist mothers in overcoming breastfeeding problems [33].

In addition to identifying methods to reach more individual newborns, it is also important to study PNC coverage at a subnational level to enable countries to address geographic inequities. In Bangladesh there is wide variation in PNC coverage. Understanding why some districts have lower coverage than others can help inform intervention strategies. Traditional practices such as seclusion of the mother and newborn may be more prevalent in some districts than others, and thus engagement with communities on the importance of PNC (whether in a facility or in the home) may be particularly helpful.

Our maps also revealed that districts with the highest PNC coverage did not necessarily have the lowest neonatal mortality and vice versa. We further explain this finding through bivariate comparisons of our key independent variables and PNC with neonatal mortality. The findings indicated significantly higher neonatal mortality when there is a PNC by day 2 check, maternal complication and delivery with a skilled birth attendant. Families in Bangladesh seem to view PNC as a service needed only when there is a problem with the mother or newborn. According to the BMMS 2010, 56% of mothers whose newborns did not receive PNC, indicated the reason was that the service was “not needed” [18]. Unfortunately, we lacked data on newborn complications, prematurity and low birth weight.

There are several limitations to this study including our inability to include certain measures including neonatal complications, four or more ANC visits and birthweight. Though we looked at bivariate associations of PNC with neonatal mortality, there could be some left censoring of the data in that some newborns could have died before they were eligible for PNC [9]. Another limitation is the measurement of PNC itself as some women may not realize their newborn is receiving a check. Direct questions on PNC without probes, as was used in the BMMS, may lead to an underestimation of coverage [34]. The BMMS did not include questions on content or quality of the PNC check, and future research is needed to develop measures on both content and quality of PNC.

Based on the findings of this study, several programmatic recommendations can be made. Both formal health workers and community health workers should provide PNC, a service essential for both for its preventative and curative elements. Particular efforts are needed to reach rural and poor women. Bangladesh has already taken relevant steps to reduce neonatal mortality by developing a National Newborn Health Strategy, which includes PNC provided by community health workers as a national health sector program approach [35]. Important considerations for such an approach are ensuring community health workers have the proper training, supplies and supervision to conduct their work. Community health workers also need to be notified of both facility and home births so that more newborns can be reached [36]. In addition to supporting community health workers, efforts are needed to increase the quality of PNC services in health facilities and to educate families on recognizing the value of PNC for seemingly healthy newborns as well as for sick newborns.
